# Facile Fabrication of Starch–Zein Core–Shell Microparticles by Antisolvent Precipitation for Reducing Starch Digestibility

**DOI:** 10.3390/foods15060996

**Published:** 2026-03-11

**Authors:** Chaofan Wang, Na Ji, Qingjie Sun

**Affiliations:** 1 College of Economics and Management, Beijing University of Technology, Beijing 100124, China; 2 College of Food Science and Engineering, Qingdao Agricultural University, Qingdao 266109, China; 3 Qingdao Special Food Research Institute, Qingdao 266109, China

**Keywords:** core–shell structure, antisolvent precipitation, zein, starch digestion

## Abstract

This study aimed to slow down starch digestion by encapsulating the starch granule within a firm zein shell via solvent-exchange-induced zein deposition. The zein shell adhered tightly to the granule surface and the shell thickness increased with increasing zein concentration. The average shell thickness of microparticles produced with zein (1%, 2%, and 3% *w*/*v*) was 0.54 μm, 0.97 μm, and 1.63 μm, respectively. Thicker zein shells acted as a mechanical barrier limiting heat transfer and water penetration, thus significantly affecting the starch digestibility. The in vitro simulated digestion experiment indicated that CS-3% zein microparticles exhibited an approximately 19-fold higher resistant starch (RS) content compared with native corn starch. These findings demonstrated the potential of the zein acting as a shell material in developing delivery system for controlled starch digestion. Additionally, this study validated antisolvent precipitation as an effective method to construct hydrophilic core/hydrophobic shell delivery systems to encapsulate unstable and hygroscopic compounds.

## 1. Introduction

Starch, one of the primary carbohydrates, provides the energy to maintain daily activities and metabolism in the human body [1]. Starch-based foods are usually rich in rapidly digestible starch (RDS). During digestion, RDS is rapidly broken down to glucose, leading to excessive postprandial glucose spikes. Long-term consumption of RDS-rich diets is associated with increased risks of obesity, chronic metabolic diseases, and irreversible organ dysfunction [2]. Consequently, extensive studies have been carried out to develop novel starch-based foods with low digestibility to prevent acute postprandial blood glucose fluctuations.

Recent studies have reported a promising method for encapsulating starch granules using polysaccharides and proteins to hinder the access of water and digestive enzymes during digestion [3]. The most frequently used encapsulation method involves introducing droplets composed of polysaccharides and starch into a crosslinking agent to form a gel network [3]. However, some researchers have found that a large amount of starch is exposed on the exterior of polysaccharides/starch microparticles due to the random distribution of starch within polysaccharide-starch composite droplets during encapsulation [4]. These starch granules that are not completely encapsulated within the polysaccharide matrix can be quickly digested [5]. Zein, a GRAS-approved hydrophobic corn protein, is frequently employed as a shell material for starch-based microparticles because of its self-assembly behavior and strong moisture-barrier properties [4]. Unlike polysaccharide matrices that often fail to fully envelop starch granules, zein can form continuous shells capable of providing complete surface coverage [5]. For example, Lin et al. (2025) [5] demonstrated that at sufficiently high concentrations (e.g., 5%), zein forms a complete hydrophobic coating on gellan gum–corn starch beads. Compared with uncoated beads, the slowly digestible starch (SDS) proportion of gellan gum–corn starch beads encapsulated by a zein shell increased by approximately 14.43% [5].

Native zein exhibits poor affinity toward starch granules because of thermodynamic incompatibility between hydrophobic protein domains and hydrophilic starch surfaces, resulting in weak adsorption rather than compact shell formation [6]. Therefore, the current methods reported in the literature for preparing starch-based core–shell microparticles with zein shells involve the initial fabrication of polysaccharides/starch microparticles, followed by the formation of zein shells on the surface of these microparticles via electrostatic interactions with polysaccharides [5]. However, starch-based core–shell microparticles with zein shells, in the form of encapsulated single starch granules, have not yet been reported.

Chen et al. (2019) found that some amino acid residues of zein, such as asparagine, show high affinity toward amylose [7]. Amylose–zein interactions can increase the structural stability of amylose [7]. As described by previous reports, the structural stability of amylose could affect the thermal stability and hydration properties [8], thus indirectly affecting the starch digestibility [9,10]. Therefore, researchers have been interested in the method to retard starch digestion through enhanced zein–starch interactions. One of the promising ways involves exposing hydrophilic amino acid residues of zein by thermal treatment above 90 °C [7,11,12] and increasing the accessibility of amylose by preparing granular cold-water swelling starch [13,14,15].

Based on the research mentioned above, we designed a novel approach for simply fabricating starch–zein core–shell microparticles by antisolvent precipitation. The method involved dripping the aqueous suspension of swollen corn starch granules into an ethanol–water solution containing thermal-treated zein at room temperature. At the granule surface, outward diffusion of water and inward migration of ethanol reduced local zein solubility and triggered its precipitation into a shell tightly adhered to the granule surface. Our previous study has demonstrated that the catalytic effects of Transglutaminase (TGase) on the crosslinking of zein improved the compactness of the zein shells [9]. Therefore, TGase was employed in this study to reinforce the zein shell.

The principal aim of the research was to design a facile method for encapsulating starch granules within a firm zein shell that adheres tightly to the granule surface to retard starch digestion. The microstructure, particle size distribution, thermal stability, and hydration properties of starch–zein core–shell microparticles produced with different concentrations of zein were investigated. In vitro digestion assays were performed to find out the impact of zein concentration on the digestibility of starch–zein core–shell microparticles. These findings could provide a new strategy for designing a potential delivery system for controlled starch digestion. Additionally, this study validated antisolvent precipitation as an effective method to construct hydrophilic core/hydrophobic shell delivery systems to encapsulate unstable and hygroscopic compounds.

## 2. Materials and Methods

### 2.1. Materials and Chemical Reagents

Corn starch was purchased from Zhucheng Xingmao Corn Development Co., Ltd. (Liaocheng, China). TGase (1000 U/g), zein, pancreatin (8× USP), α-glucosidase (260 U/mL), rhodamine B, and Nile blue were sourced from Merck & Co., Inc. (Rahway, NJ, USA).

### 2.2. Preparation of Starch–Zein Core–Shell Microparticles

The processing technology of swollen corn starch granules was adapted from the technique of Kaveh et al. [16] to ensure controlled granule swelling. Briefly, 3 g of corn starch was dispersed in 35 g of 40% (*v*/*v*) ethanol–water solution with continuous stirring until a uniform suspension was obtained. The suspension was mixed with sodium hydroxide solution (2.5 M, 12 g) by stirring for 15 min to induce granule swelling and then neutralized with 2.5 M hydrochloric acid prepared in absolute ethanol. Subsequent centrifuging and washing were conducted to obtain swollen corn starch granules. Ultrapure water was added to swollen corn starch granules until the volume of the prepared starch suspension reached 30 mL.

Separate zein samples (1, 2, and 3 g) were each fully solubilized in 100 mL of a 70% ethanol–water solution, followed by heating the solutions above 90 °C for 30 min to obtain thermal-treated zein. To obtain the starch–zein core–shell microparticles, 30 mL of the prepared starch suspension was added slowly (approximately 0.5 mL/min) to 45 mL of an ethanol–water solution containing thermal-treated zein. The antisolvent precipitation process lasted about 2–3 h under continuous stirring at a rate of 100 rpm to allow solvent exchange and zein deposition. Subsequent centrifuging and washing were conducted to completely remove ethanol and unabsorbed zein.

**Figure 1:**
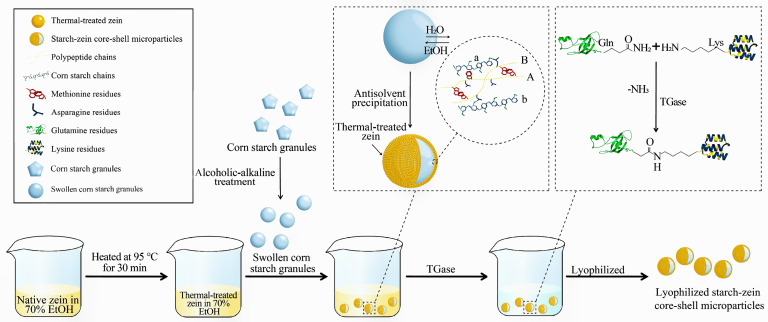
The preparation process of starch–zein core–shell microparticles.

The crosslinking of zein was adapted from the technique reported in the previous literature [17,18]. TGase (4.0% of zein weight) was solubilized in 25 mL of ultrapure water by stirring at 38 °C, followed by mixing the enzyme solution and the starch–zein core–shell microparticles. After 2 h of reaction at 38 °C, subsequent centrifuging and washing were conducted to completely remove the zein that adsorbed weakly onto the granule surface. The microparticles produced with zein (1%, 2%, and 3% *w*/*v*) were subsequently freeze-dried and denoted as CS-1%, CS-2%, and CS-3% zein microparticles, respectively. The unshelled starch control was prepared following the same procedure illustrated in Figure 1 without zein.

### 2.3. Optical Microscopy

The suspension of samples prepared with ultrapure water was placed under an optical microscope (CCD TP510, Optec International Inc., Carlsbad, CA, USA) for observation with a 40× objective.

### 2.4. Fluorescence Microscopy

The zein shell was labeled with rhodamine B by mixing 2 g of starch–zein core–shell microparticles and 25 mL of rhodamine B aqueous solution (0.001%, *w*/*v*) with continuous stirring for 1 min. Subsequent centrifuging and washing were conducted to completely remove residual rhodamine B. The zein shell of starch–zein core–shell microparticles was visualized using a fluorescence microscope (BX53, Olympus Corp., Shinjuku City, Japan). The magnification and the excitation wavelength were set as 400× and 530–550 nm, respectively.

### 2.5. Confocal Laser Scanning Microscopy (CLSM)

Briefly, 2 g of starch–zein core–shell microparticles were incubated with Nile blue aqueous solution (0.001%, *w*/*v*) with continuous stirring for 1 min to label the zein shell. Subsequent centrifuging and washing were conducted to completely remove residual Nile blue. CLSM (TCS SP5 II, Leica Microsystems Inc., Wetzlar, Germany) was used to visualize the zein shell of starch–zein core–shell microparticles. The magnification and the excitation wavelength were set as 400× and 633 nm, respectively. The average shell thickness was measured using the software provided with the microscope.

### 2.6. Surface Morphology

The surface morphology of samples (native corn starch and CS-1%, CS-2%, and CS-3% zein microparticles) was visualized using a JSM-7500F scanning electron microscope (JEOL, Tokyo, Japan). The magnification and the accelerating voltage were set as 3000× and 5 kV, respectively.

### 2.7. Particle Size Distribution (PSD)

PSD was evaluated using a laser particle size analyzer (Nano ZS90, Malvern Instruments Ltd., Malvern, UK). Before measurement, the suspension of samples prepared with ultrapure water was dispersed by ultrasonic treatment (100 W, 10 s).

### 2.8. Crystal Structure

Lyophilized samples were ground into powders before XRD measurement. XRD patterns were recorded using a D8 Advance X-ray diffractometer (Bruker Corp., Bremen, Germany). Analyses were carried out using the Cu Kα radiation (λ = 1.5405 Å). The scan range and the scan rate were set as 4–40° and 2°/min, respectively.

### 2.9. Thermal Stability

Thermal transitions were evaluated using a differential scanning calorimeter (Mettler-Toledo International Inc., Greifensee, Switzerland). 4.00 mg (dry weight) of sample and 8 μL of ultrapure water were mixed in a hermetic crucible. After storing overnight to ensure uniform hydration, samples were heated from 25 to 125 °C at a constant rate of 10 °C/min.

### 2.10. Hydration Properties

Separate suspensions, each consisting of 200 mg (Ws, dry weight) of sample and 20 mL of ultrapure water, were placed at 55, 65, 75, 85, and 95 °C for 30 min. Subsequent cooling to 25 °C and centrifuging were conducted to obtain supernatant and precipitate. The supernatant was dried to a constant weight (Wa) to determine the mass of solubilized starch. The weight of precipitate was precisely measured and recorded (Wb) to determine swelling capacity. Water solubility (WS, %) and swelling power (SP, g/g) were calculated using the following equations:

(1) WS = Wa/Ws × 100

(2) SP = (Wb × 100)/Ws × (100 − WS)

### 2.11. In Vitro Simulated Digestion Experiment

As described by Englyst et al. [19], the suspension consisting of 3 g of pancreatin and 20 mL of ultrapure water was vortexed for 5 min to obtain supernatant. The enzyme solution was composed of 1.1 mL of α-glucosidase and 15 mL of supernatant. Exactly 200 mg (dry weight) of sample was dispersed in sodium acetate buffer (18 mL, pH was adjusted to 5.20) and heated at 100 °C for 0.5 h to simulate the cooking process. After cooling down to 37 °C, the mixture was reacted with 2 mL of enzyme solution with constant shaking to simulate gastrointestinal peristalsis. Amounts of 0.1 mL of the mixture were collected at 0, 20, 40, 60, 80, 100, and 120 min and were reacted with 0.9 mL of 90% ethanol–water solution to denature digestive enzymes. Subsequent centrifuging was conducted to obtain the supernatant for measuring glucose content. The contents of RDS (%), SDS (%), and RS (%) were calculated using the following equations:

(3) RDS = (C20 − C0) × 0.9/Cs × 100

(4) SDS = (C120 − C20) × 0.9/Cs × 100

(5) RS = (1 − RDS − SDS) × 100

C0, C20, and C120 represent the released glucose content at 0, 20, and 120 min, respectively. Cs represents the starch content of samples.

### 2.12. Data Analysis

Data were analyzed by SPSS V.22 software (SPSS Inc., Chicago, IL, USA) and presented as mean values with standard deviations. Statistical comparison was performed using analysis of variance (ANOVA) and Duncan’s test. *p* < 0.05 was considered statistically significant.

## 3. Results and Discussion

### 3.1. Formation Mechanism of Starch–Zein Core–Shell Microparticles

Figure 1 shows the processing technologies of starch–zein core–shell microparticles. The swollen corn starch granules were fabricated by alcoholic-alkaline treatment, in which alkali was used to promote starch swelling by enhancing the negative charge repulsion on starch molecules. The main function of ethanol was to prevent excessive starch swelling by forming V-complexes with the single helical structure and to induce the entanglement of dissociated starch chains [14,15]. After ethanol evaporation, the resulting internal space inside the granule acted as a micro channel for water [15]. The formation of core–shell structure can be attributed to solvent-exchange-induced antisolvent precipitation of zein. The process involved dripping the prefabricated aqueous suspension of swollen corn starch granules into an ethanol–water solution containing thermal-treated zein. At the granule surface, outward diffusion of water and inward migration of ethanol reduced local zein solubility and triggered its precipitation into a shell tightly adhered to the granule surface.

The potential starch–zein interactions are shown in Figure 1. Asparagine residues (deep blue) are located at the polypeptide chains (denoted as A and B, yellow) of zein. These residues act as binding sites interacting with chains of starch molecules (denoted as a and b, pale blue) [11]. Polypeptide chains interact with each other via hydrophobic interaction through their methionine residues (red). Additionally, polypeptide chains could connect two starch chains via asparagine [11].

TGase was employed to catalyze covalent crosslinking within the zein matrix to further reinforce the zein shell. As shown in Figure 1, TGase induced the production of isopeptide bonds by catalyzing the transfer reactions of acyl groups that occur between glutamine (Gln) and lysine (Lys) [20]. The catalytic effects of TGase on the crosslinking of zein improved the compactness of the zein shells.

### 3.2. Morphological Analysis

**Figure 2:**
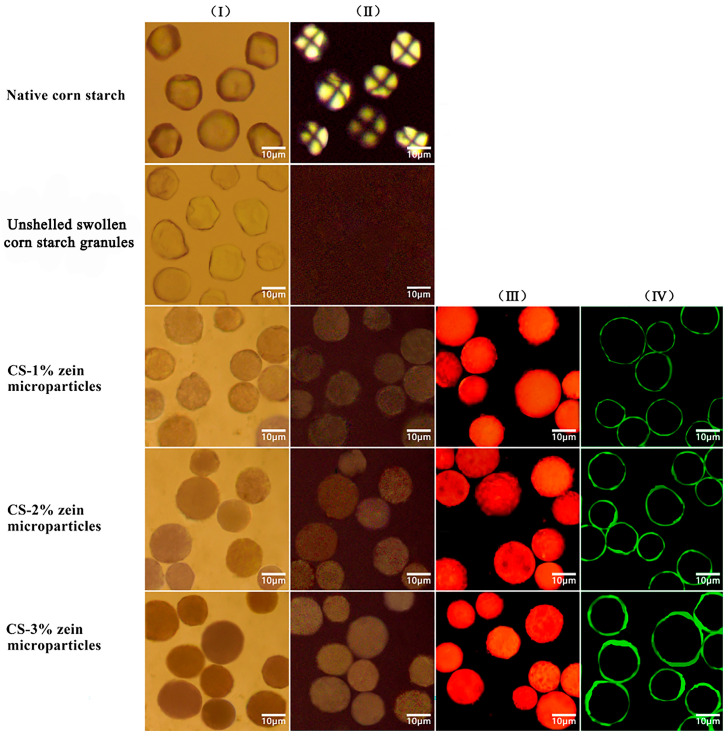
The images of particle morphology captured by optical microscope (columns I and II), fluorescence microscope (column III), and confocal scanning laser microscope (column IV).

As shown in Figure 2, optical microscopy, fluorescence microscopy, and CLSM are used to observe the particle morphology. Native corn starch was mostly polygonal with sharply defined edges (column I). Due to the existence of ordered microcrystalline structure within starch molecules, these granules exhibited a characteristic Maltese cross morphology at the center of hilum (column II) when observed under polarized light. Following alcoholic-alkaline treatment, the granules were swollen into nearly spherical structures while remaining unruptured (column I). In addition, the disappearance of Maltese cross morphology under polarized light (column II) indicated the disruption of crystalline domains and partial gelatinization during swelling. The optical microscopy showed that the starch–zein core–shell microparticles were approximately spherical and almost opaque (column I). The opacity of core–shell microparticles increased with the increasing zein concentration, confirming the gradual deposition of zein at the surface of starch granules.

Fluorescence microscopy revealed additional details demonstrating that the swollen starch granules were fully enveloped within the zein shells. Unlike swollen granules that showed no fluorescence, the starch–zein core–shell microparticles labeled with rhodamine B exhibited integral spherical fluorescence (column III), which indicated that the fluorescence was emitted from the zein shells.

We performed CLSM visualization on microparticles labeled with Nile blue to further confirm the core–shell structure. As displayed in Figure 1 (column IV), the zein shell was clearly visualized, and the shell thickness increased with increasing zein concentration. The average shell thickness of microparticles produced with zein (1%, 2%, and 3% *w*/*v*) was 0.54 μm, 0.97 μm, and 1.63 μm, respectively. This trend indicated that zein concentration was closely related to the resulting shell thickness.

**Figure 3:**
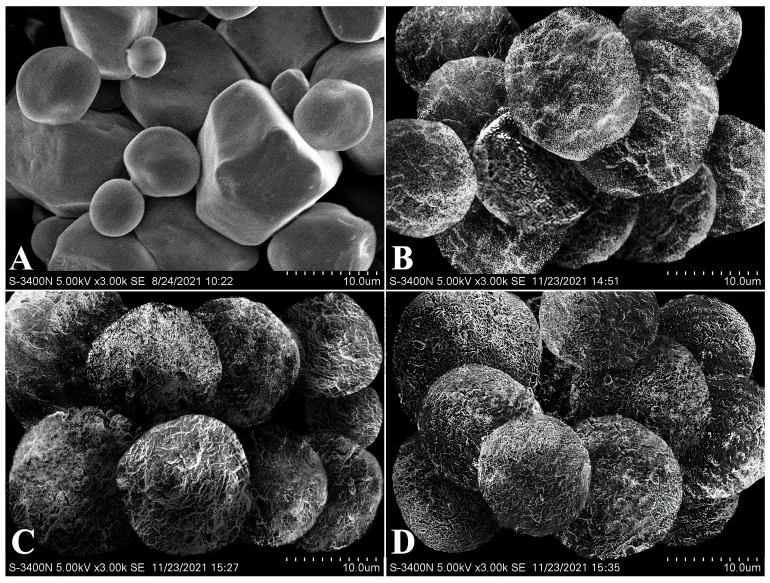
Scanning electron microscopy images of native corn starch (**A**) and starch–zein core–shell microparticles produced with zein concentrations of 1% (**B**), 2% (**C**), and 3% *w*/*v* (**D**), respectively.

High-resolution images of the sample surface captured by scanning electron microscope are shown in Figure 3. Native corn starch granules exhibited a characteristic polygonal morphology with sharply defined edges and relatively smooth surfaces. Following alcoholic-alkaline treatment, the granules exhibited an approximately spherical morphology due to alkali-induced granule swelling. Compared with native starch, the core–shell microparticles showed much rougher surfaces due to the zein deposition. The production of zein shells might be carried out through the nucleation and extension of zein molecular clusters at the granule surface [21]. The surface roughness and shell thickness of starch–zein core–shell microparticles increased with increasing zein concentration. A case in point is that the CS-3% microparticles exhibited the roughest surfaces (Figure 3) and the thickest shells (Figure 1). These results demonstrated that the shell development was highly dependent on the zein concentration.

### 3.3. Particle Size Distribution (PSD)

**Figure 4:**
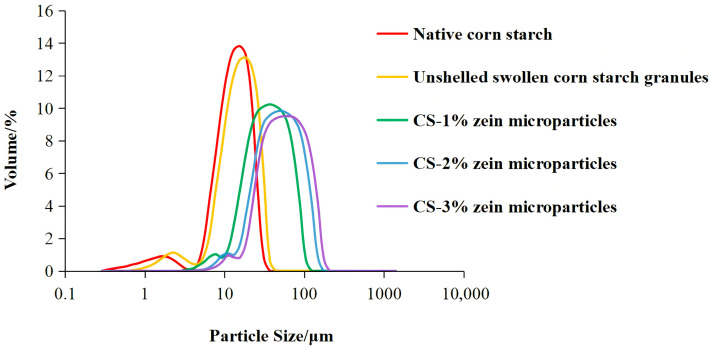
Particle size distribution of samples.

As shown in Figure 4, the particle sizes of the samples were primarily distributed in two ranges: 0–10 µm and 10–100 µm. Table 1 shows the characteristic values of PSD. Due to water permeation during starch gelatinization, unshelled swollen corn starch granules showed larger volume mean diameter d(4,3) and wider PSD compared with native corn starch. Furthermore, starch–zein core–shell microparticles showed significantly larger d(4,3) compared with other samples. The PSD of starch–zein core–shell microparticles (3.27–176.00 µm) was wider than that of the native corn starch (0.34–31.11 µm) and the unshelled swollen corn starch granules (0.69–37.00 µm). It is possible that the zein shells of some adjacent microparticles fused due to the catalytic effects of TGase, which induced the production of isopeptide bonds both intramolecularly and intermolecularly [20]. The d(4,3) and the PSD of starch–zein core–shell microparticles increased with increasing zein concentration. This trend can be attributed to thicker zein shells produced with higher zein concentration. The thickness of the zein shell could affect the thermal stability and enzymatic accessibility, thus indirectly affecting the digestibility discussed in later sections.

### 3.4. Crystal Structure

**Figure 5:**
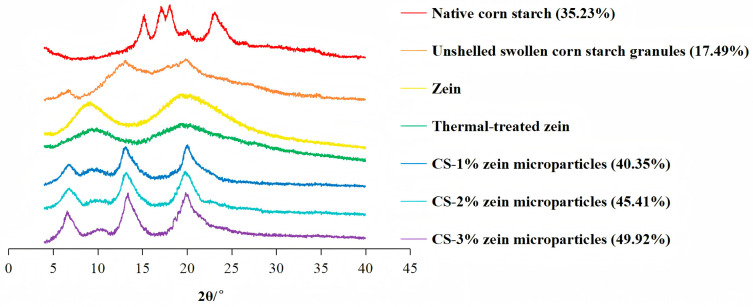
X-ray diffraction patterns of samples. The relative crystallinity is provided in parenthesis.

As illustrated in Figure 5, the main peaks of native corn starch appeared at 15.2°, 17.2°, 18°, and 23.1° (2θ), which were the typical characteristics of A-type crystal structure [22]. Unshelled swollen corn starch granules showed a V-type crystal structure, the main XRD peaks of which appeared at 6.9°, 13.1°, and 19.8° (2θ). It can probably be explained by the molecular rearrangement and the disruption of starch crystallites [23].

The peak of zein at 9.9° (2θ) characterizes the right-handed helical structure formed by polypeptide chains spiraling upwards around the central axis, while the peak of zein at 20.1° (2θ) characterizes the sheet-like structure formed by polypeptide chains arranged in parallel or antiparallel fashion [24]. After heat treatment, the peak intensity of two peaks was reduced significantly, which indicated the disintegration of the original crystal structure and the reduced molecular orderliness within zein [24], thus improving the affinity of zein toward starch.

Starch–zein core–shell microparticles showed both the characteristic peaks of zein presented at 10° (2θ) and the same V-type characteristic peaks as unshelled swollen corn starch granules, indicating the formation of zein–starch interactions. The peak intensity and crystallinity of starch–zein core–shell microparticles increased with increasing zein concentration due to the peak superposition and the enhanced polypeptide-starch chain connections caused by the aggregation of precipitated zein on the granule surface [25]. Such structural alterations could increase the structural stability of starch chains and hinder the access of water and digestive enzymes, thus affecting the thermal stability and the digestibility discussed in later sections.

### 3.5. Thermal Stability and Hydration Properties

The thermal parameters of samples are provided in Table 2. Due to the disruption of native crystalline structures caused by alcoholic-alkaline treatment, unshelled swollen corn starch granules were easily gelatinized. Thus, they showed significantly lower thermal transition temperatures and enthalpy change compared with native corn starch, indicating a reduction in thermal energy used to unwind the entanglement of starch chains.

Starch–zein core–shell microparticles showed higher thermal transition temperatures compared with native corn starch. The underlying reason is that the barrier formed by zein matrix could limit heat transfer and prevent water penetration into the starch crystalline structure during heating [26,27]. Thus, the resistance to heat of starch–zein core–shell microparticles was better than that of native corn starch. Similar phenomena have also been reported in other starch–protein systems, such as corn starch/pine kernel protein/egg white protein complexes [28] and pea starch/fava bean protein complexes [29]. Furthermore, starch–zein core–shell microparticles showed lower enthalpy change compared with native corn starch, and the value decreased with increasing shell thickness. This trend is explained by the enhanced solidity of the thicker zein shells, which effectively prevented the gelatinization of some microparticles. Thus, these starch–zein core–shell microparticles contributed none or little endothermic enthalpy during the DSC heating. Such enhanced solidity could also contribute to the limited starch swelling and digestibility discussed in later sections.

**Figure 6:**
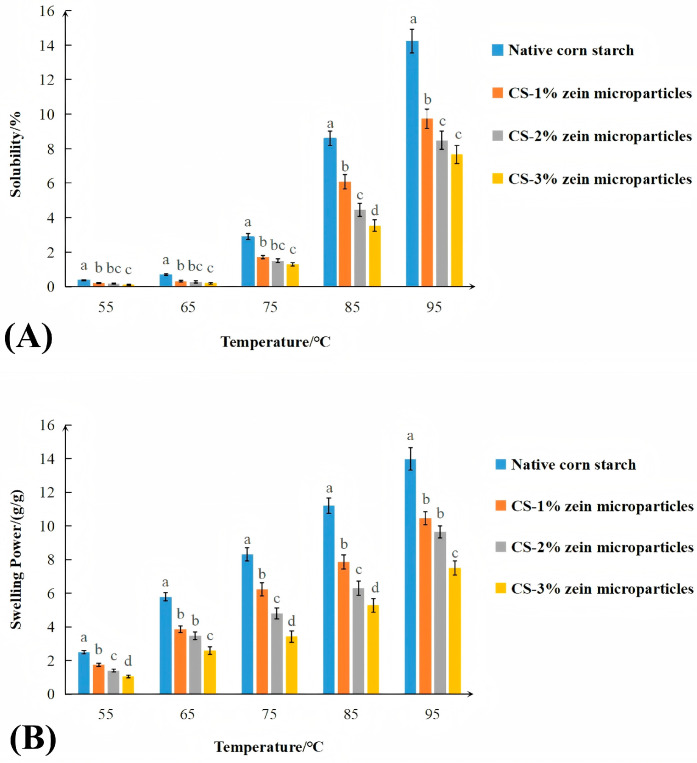
Water solubility (**A**) and swelling power (**B**) of samples. Values in the same column with different letters are significantly different (*p* < 0.05).

Previous studies have demonstrated that starch, after removal of endogenous proteins, showed higher swelling properties and digestibility compared with native corn starch [30]. Correspondingly, our findings suggested the inhibitory effect of exogenous zein on starch swelling. As shown in Figure 6, both WS and SP exhibited an increase with increasing temperature. Unshelled swollen granules showed significantly higher WS and SP values compared with native corn starch, suggesting that the disrupted crystalline structures facilitated water penetration.

These hydration parameters were lower in starch–zein core–shell microparticles than in unshelled swollen granules and decreased further with increasing zein concentration. This trend could be explained by the zein shell acting as a barrier that limited water penetration and starch swelling. In addition, the hydrophobicity of zein could also inhibit the starch–water interactions [26,27].

In summary, DSC and hydration results indicated that thicker zein shells act as a mechanical barrier limiting heat transfer and water penetration. This restricted water and heat conduction led to the reduced gelatinization and the suppressed structural disintegration, thus contributing to lower enzymatic digestibility. However, additional research is necessary to demystify the underlying physicochemical mechanisms.

### 3.6. In Vitro Digestibility and Digestion-Induced Morphological Changes

**Figure 7:**
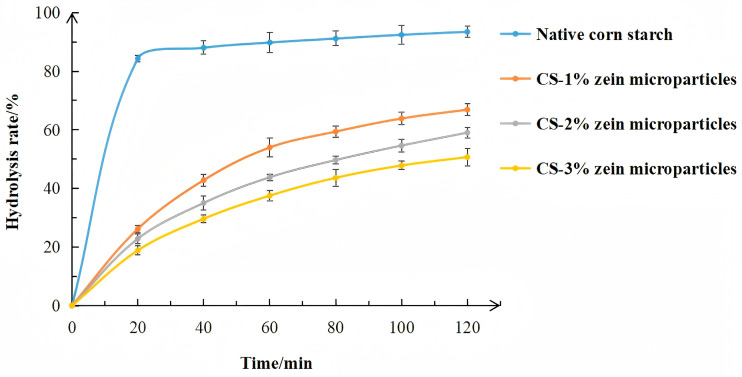
Glucose hydrolysis curves of cooked samples.

The glucose hydrolysis rate of cooked samples is shown in Figure 7. After being cooked for 30 min, the glucose hydrolysis rate of native corn starch reached over 80% within the first 20 min of enzymatic hydrolysis, suggesting its large RDS proportion (>80%). The starch–zein core–shell microparticles were digested slowly due to the zein shell acting as a barrier that shielded starch from enzymatic digestion. The glucose hydrolysis rate of the microparticles decreased with increasing zein concentration. For example, during the first 20 min of enzymatic hydrolysis, the glucose hydrolysis rate of CS-1% zein microparticles was 26.16%, while that of the CS-3% microparticles was only 18.83%. These results confirmed that zein concentration acts as a key factor controlling the early-stage enzymatic hydrolysis (within the first 20 min) and the RDS proportion. During this stage, thicker zein shells exerted a stronger resistance to enzymatic penetration.

The SDS proportion of starch–zein core–shell microparticles decreased with increasing zein concentration, indicating sustained resistance to enzymatic hydrolysis during the intermediate-stage hydrolysis process (20–120 min). CS-3% zein microparticles exhibited the lowest SDS proportion. This trend confirmed that thicker zein shells delayed enzymatic access, thus prolonging the hydrolysis process.

Table 3 shows the content of RDS, SD, and RS of samples. The RDS content of native corn starch reached 87.96%, and the RS content was only 2.48%. In contrast, the RDS content was 27.05% and 19.36% for CS-1% zein microparticles and CS-3% zein microparticles, respectively. More importantly, compared with native corn starch, CS-1% zein microparticles and CS-3% zein microparticles exhibited approximately 12-fold and 19-fold higher RS content, respectively.

**Figure 8:**
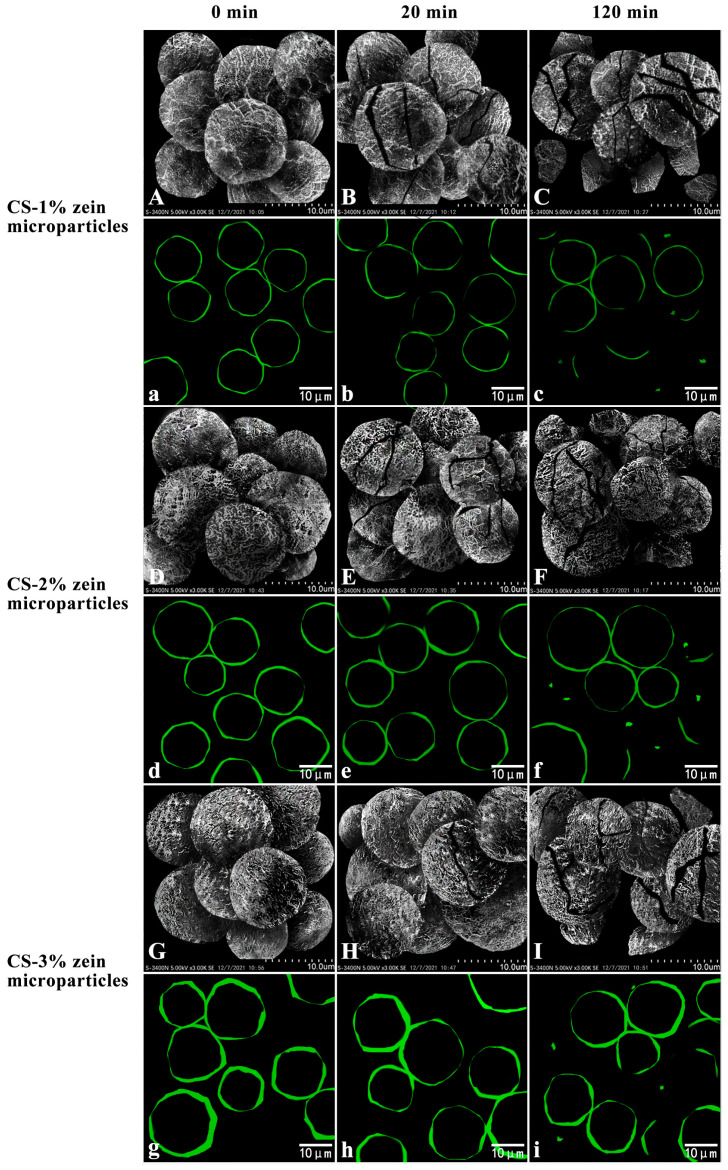
Morphological changes in starch–zein core–shell microparticles during in vitro digestion. Scanning electron microscopy (**A**–**I**) and confocal laser scanning microscopy (**a**–**i**) images of microparticles produced with zein concentrations of 1% (**A**–**C**,**a**–**c**), 2% (**D**–**F**,**d**–**f**), and 3% *w*/*v* (**G**–**I**,**g**–**i**) after 0, 20, and 120 min of digestion, respectively.

SEM and CLSM were used to analyze the morphological changes in starch–zein core–shell microparticles during starch digestion. As shown in Figure 8, after being cooked for 30 min, the starch–zein core–shell microparticles were unruptured (column I). After the microparticles were digested for 20 min, several cracks were observed on their surface (column II). Compared with CS-1% zein microparticles (Figure 8B,b) and CS-2% zein microparticles (Figure 8E,e), CS-3% zein microparticles (Figure 8H,h) exhibited fewer cracks and higher structural stability. After 120 min of digestion, the number of cracks on the surface of microparticles increased (column III). Several microparticles were disintegrated into fragments and chunks (column III). Compared with CS-1% zein microparticles (Figure 8C,c) and CS-2% zein microparticles (Figure 8F,f), CS-3% zein microparticles (Figure 8I,i) exhibited fewer cracks, fragments, and chunks, indicating that the resistance to enzymatic hydrolysis of starch–zein core–shell microparticles increased with increasing shell thickness.

Taken together, the experimental results of digestibility and morphological changes demonstrated that thickness of the zein shells determined the water penetration and enzymatic accessibility. Thicker zein shells exerted a stronger resistance to heat transfer and enzymatic penetration, resulting in lower RDS, higher SDS, and significantly elevated RS. These findings provided direct mechanistic evidence that antisolvent-precipitated zein shells can be strategically engineered to control starch digestion.

## 4. Conclusions

This study aimed to design a facile method to retard starch digestion by encapsulating the starch granule within a firm zein shell that adhered tightly to the granule surface. The shell thickness increased with increasing zein concentration. The average shell thickness of microparticles produced with zein (1%, 2%, and 3% *w*/*v*) was 0.54 μm, 0.97 μm, and 1.63 μm, respectively. Thicker zein shells acted as a mechanical barrier limiting heat transfer and water penetration, thus significantly affecting the starch digestibility. The in vitro simulated digestion experiment indicated that CS-3% zein microparticles exhibited an approximately 19-fold higher resistant starch (RS) content compared with native corn starch. This discovery will provide a new strategy for designing a potential delivery system for the controlled digestion of starch. Additionally, these findings have theoretical significance for designing hydrophilic core/hydrophobic shell delivery systems to encapsulate both nutraceuticals and pharmaceuticals that are easily destroyed under gastrointestinal conditions.

## Figures and Tables

**Table 1:** Particle size distribution parameters of samples.

Sample	d(4,3)/μm	d(0.1)/μm	d(0.5)/μm	d(0.9)/μm	Particle Size Range/μm
Native corn starch	12.44 ± 0.06 ^e^	5.74 ± 0.07 ^e^	12.03 ± 0.08 ^e^	20.39 ± 0.03 ^e^	0.34–31.11
Unshelled swollen corn starch granules	14.83 ± 0.05 ^d^	6.61 ± 0.09 ^d^	14.13 ± 0.07 ^d^	24.75 ± 0.03 ^d^	0.69–37.00
CS-1% zein microparticles	36.09 ± 0.07 ^c^	14.14 ± 0.04 ^c^	31.78 ± 0.08 ^c^	65.55 ± 0.06 ^c^	3.27–104.70
CS-2% zein microparticles	50.15 ± 0.09 ^b^	19.18 ± 0.07 ^b^	43.51 ± 0.05 ^b^	92.55 ± 0.08 ^b^	4.62–148.00
CS-3% zein microparticles	59.09 ± 0.03 ^a^	22.62 ± 0.06 ^a^	50.89 ± 0.06 ^a^	110.16 ± 0.06 ^a^	5.50–176.00

Data were presented as mean values with standard deviations. Values in the same column with different letters are significantly different (*p* < 0.05).

**Table 2:** Thermal transition temperatures and enthalpy change in samples.

Sample	To/°C	Tp/°C	Tc/°C	ΔH/(J/g)
Native corn starch	65.24 ± 0.27 ^d^	70.02 ± 0.28 ^d^	74.88 ± 0.12 ^d^	−12.06 ± 0.07 ^a^
Unshelled swollen corn starch granules	60.47 ± 0.36 ^e^	67.82 ± 0.22 ^e^	73.17 ± 0.20 ^e^	−3.73 ± 0.19 ^d^
CS-1% zein microparticles	74.17 ± 0.09 ^c^	77.31 ± 0.11 ^c^	80.66 ± 0.16 ^c^	−4.75 ± 0.13 ^b^
CS-2% zein microparticles	76.67 ± 0.18 ^b^	79.50 ± 0.20 ^b^	82.85 ± 0.16 ^b^	−3.95 ± 0.07 ^c^
CS-3% zein microparticles	81.48 ± 0.11 ^a^	84.00 ± 0.09 ^a^	87.70 ± 0.13 ^a^	−2.66 ± 0.10 ^e^

Data were presented as mean values with standard deviations. Values in the same column with different letters are significantly different (*p* < 0.05).

**Table 3:** The content of rapidly digestible starch (RDS), slowly digestible starch (SDS), and resistant starch (RS) of samples.

Sample	RDS/%	SDS/%	RS/%
Native corn starch	87.96 ± 1.12 ^a^	9.56 ± 2.06 ^d^	2.48 ± 1.96 ^d^
CS-1% zein microparticles	27.05 ± 1.32 ^b^	42.55 ± 1.90 ^a^	30.39 ± 1.90 ^c^
CS-2% zein microparticles	23.63 ± 1.81 ^c^	37.86 ± 1.51 ^b^	38.51 ± 1.95 ^b^
CS-3% zein microparticles	19.36 ± 1.75 ^d^	33.27 ± 3.13 ^c^	47.36 ± 3.34 ^a^

Data were presented as mean values with standard deviations. Values in the same column with different letters are significantly different (*p* < 0.05).

## Data Availability

The original contributions presented in the study are included in the article, further inquiries can be directed to the corresponding author.

## References

[1] Freitas D., Lazaridou A., Duijsens D., Kotsiou K., Corbin K.R., Alongi M., Perez-Moral N., Simsek S., Nehir El S., Gwala S. (2025). Starch digestion: A comprehensive update on the underlying modulation mechanisms and its in vitro assessment methodologies. Trends Food Sci. Tech..

[2] Yin J., Cheng L., Hong Y., Li Z.F., Li C.M., Ban X.F., Zhu L., Gu Z.B. (2025). Slow starch digestibility promotes the development of favorable feeding behaviors and metabolic health in mice. Food Res. Int..

[3] Kraithong S., Theppawong A., Huang R. (2023). Encapsulated starch characteristics and its shell matrix mechanisms controlling starch digestion. Food Chem..

[4] Tadele D.T., Islam M.S., Mekonnen T.H. (2025). Zein-based nanoparticles and nanofibers: Co-encapsulation, characterization, and application in food and biomedicine. Trends Food Sci. Tech..

[5] Lin Z.W., Li W.X., Zhang C.C., Zhan L.J., He X.Y., Qin Y., Sun Q.J., Ji N. (2025). Novel one-step method to construct gellan gum-zein core-shell structured starch beads for regulating starch digestion. Food Chem..

[6] Han L.Y., Zhu J.Z., Jones K.L., Yang J.X., Zhai R.Y., Cao J.J., Hu B. (2024). Fabrication and functional application of zein-based core-shell structures: A review. Int. J. Biol. Macromol..

[7] Chen X., Cui F.H., Zi H., Zhou Y.C., Liu H.S., Xiao J. (2019). Development and characterization of a hydroxypropyl starch/zein bilayer edible film. Int. J. Biol. Macromol..

[8] Xu H.X., Zhang S.B., Yu W.W. (2022). Revealing the mechanism beneath the effects of starch-amino acids interactions on starch physicochemical properties by molecular dynamic simulations. Food Hydrocoll..

[9] Wang C.F., Qin K.L., Sun Q.J., Qiao X.G. (2022). Preparation of natural food-grade core-shell starch/zein microparticles by antisolvent exchange and transglutaminase crosslinking for reduced digestion of starch. Front. Nutr..

[10] Yang Z.L., Zhang Y.Y., Wu Y.W., Ouyang J. (2023). Factors influencing the starch digestibility of starchy foods: A review. Food Chem..

[11] Lopez-Baron N., Gu Y.C., Vasanthan T., Hoover R. (2017). Plant proteins mitigate in vitro wheat starch digestibility. Food Hydrocoll..

[12] Wang Y.Y., Xu Z. (2025). Interactions of the molecular assembly of protein as encapsulation materials: A review. Food Chem..

[13] Li J.X., Capuano E., Tong L.T. (2025). Transformation of native starch into V-type granular starch through ethanol-aqueous heat treatment and its swelling behavior in cold water. Carbohydr. Polym..

[14] Dries D.M., Gomand S.V., Goderis B., Delcour J.A. (2014). Structural and thermal transitions during the conversion from native to granular cold-water swelling maize starch. Carbohydr. Polym..

[15] Majzoobi M., Farahnaky A. (2021). Granular cold-water swelling starch; properties, preparation and applications, a review. Food Hydrocoll..

[16] Kaveh Z., Azadmard-Damirchi S., Yousefi G., Hosseini S.M.H. (2020). Effect of different alcoholic-alkaline treatments on physical and mucoadhesive properties of tapioca starch. Int. J. Biol. Macromol..

[17] Ahammed S., Liu F., Wu J.M., Khin M.N., Yokoyama W.H., Zhong F. (2021). Effect of transglutaminase crosslinking on solubility property and mechanical strength of gelatin-zein composite films. Food Hydrocoll..

[18] Masamba K., Li Y., Hategekimana J., Zehadi M., Ma J.G., Zhong F. (2016). Evaluation of mechanical and water barrier properties of transglutaminase cross-linked zein films incorporated with oleic acid. Int. J. Food. Sci. Tech..

[19] Englyst H.N., Kingman S.M., Cummings J.H. (1992). Classification and measurement of nutritionally important starch fractions. Eur. J. Clin. Nutr..

[20] Cui H.M., Liu G.L., Padua G.W. (2016). Cell spreading and viability on zein films may be facilitated by transglutaminase. Colloids Surf. B.

[21] Ye J.P., Hu X.T., Luo S.J., Mcclements D.J., Liang L., Liu C.M. (2018). Effect of endogenous proteins and lipids on starch digestibility in rice flour. Food Res. Int..

[22] Lei M.M., Yuan H.H., Jia R.N., Huang Z.M., Yang Y., Liang Q.Y., Liu X.Y., Pan Z.L. (2025). Effects of different enzymatic hydrolysis times on the structures and properties of corn microporous starch particles and their applications in frozen dough. Food Hydrocoll..

[23] Li Q.Q., Wang Y.S., Chen H.H., Liu S., Li M. (2017). Retardant effect of sodium alginate on the retrogradation properties of normal cornstarch and anti-retrogradation mechanism. Food Hydrocoll..

[24] Han Y.L., Xu Q., Lu Z.Q., Wang J.Y. (2014). Preparation of transparent zein films for cell culture applications. Colloids Surf. B.

[25] Ferreira L.F., de Oliveira A.C.S., de Oliveira Begali D., de Sena Neto A.R., Martins M.A., de Oliveira J.E., Borges S.V., Yoshida M.I., Tonoli G.H.D., Dias M.V. (2020). Characterization of cassava starch/soy protein isolate blends obtained by extrusion and thermocompression. Ind. Crops Prod..

[26] Cabra V., Arreguin R., Vazquez-Duhalt R., Farres A. (2006). Effect of temperature and pH on the secondary structure and processes of oligomerization of 19 kDa alpha-zein. Biochim. Biophys. Acta.

[27] Sun C.X., Dai L., Liu F.G., Gao Y.X. (2016). Simultaneous treatment of heat and high pressure homogenization of zein in ethanol-water solution: Physical, structural, thermal and morphological characteristics. Innov. Food Sci. Emerg. Technol..

[28] Wang J.R., Huang W.H., Wang X.M., Zhao Y.H., Zhang L.G. (2025). Modulating the gelatinization and retrogradation characteristics of corn starch by controlling pine kernel protein/egg white protein thermal co-aggregation. Carbohydr. Polym..

[29] Ignatzy L.M., Kern K., Muranyi I.S., Alpers T., Becker T., Gola S., Schweiggert-Weisz U. (2026). Thermal, rheological, and microstructural characterization of composite gels from fava bean protein and pea starch. Food Hydrocoll..

[30] Hu N.N., Qi W.H., Zhu J.Y., Zhao F.Y., Zheng M.Z., Zhao C.B., Yan J.N., Liu J.S. (2025). Effect of endogenous protein on starch before and after post-harvest ripening of corn: Structure, pasting, rheological and digestive properties. Food Chem..

